# Gender and health – aspects of importance for understanding health and illness in the world

**DOI:** 10.3402/gha.v8.26908

**Published:** 2015-01-22

**Authors:** Ann Öhman, Malin Eriksson, Isabel Goicolea

**Affiliations:** 1 Division of Epidemiology and Global Health, Department of Public Health and Clinical Medicine, Umeå Centre for Global Health Research, Umeå University, Umeå, Sweden, Umeå Centre for Gender Studies, Umeå University, Umeå, Sweden. ann.ohman@ucgs.umu.se; 2 Division of Epidemiology and Global Health, Department of Public Health and Clinical Medicine, Umeå Centre for Global Health Research, Umeå University, Umeå, Sweden

In the call for this cluster of papers in *Global Health Action*, we included a variety of perspectives regarding gender and health to be covered, among them sexual and reproductive health and rights, gender-based violence, ageing and gender, health systems, climate change, and globalisation; all with respect to gender. From the papers that are now included, we draw the conclusion that some of these aspects are more prevalent than others. For instance, aspects of sexual and reproductive health and rights, and gender-based violence are represented by a number of papers, whereas climate change, ageing, and globalisation are not at all present. Papers oriented towards gender and health with social theories on gender are still scarce, despite the fruitful results, as some of the papers in this cluster show, for instance in Gibbs et al., Marcos et al., or Torres et al., (1–3) . We believe that this mirrors the research field of gender and health, and that new aspects are still to be covered. In this editorial, we briefly summarise and categorise the included papers and also hint at gaps and lacking perspectives when it comes to gender and health.

We view gender as a central analytical category in the studies of health. In health research at large, there is a tendency to use the concept gender as equivalent to the concept biological sex, and we can see a rise in this mix of concepts in the past decade, where the term ‘sex’ is replaced by ‘gender’ although the focus might not be on social constructions of sex but rather on biological health matters. There has for a long time been an urge for integrating theoretical gender approaches into health research (4–7) . A variety of theoretical approaches are at hand when dealing with research on gender and health and we agree with other researchers that gender is both relational and intersectorial (7, 8). When viewing gender as part of social, institutional, and structural dimensions of human lives, it also becomes evident that the links and interconnections between different power structures are at hand. It matters whether one lives as a woman, a man, or other sexual identities. All this influences health, not only at the individual level but also at all levels of human life. Gender research also problematises other expressions of sex and gender such as transsexualism, transgender, and queer perspectives. Therefore, we do not regard gender as a binary category with men and women only but also regard it as socially constructed and contextual. Gender is something we live, perform, and construct. However, as researchers in health and ill-health, we cannot disregard that the body is a biological entity (sex) as well as a socially constructed phenomenon (gender). Fausto-Sterling has been vital for theorising sex and gender and how to think about them as integral, not separate entities (9).

The papers included in this cluster that deal with *sexual and reproductive health and rights* come from different settings and take both quantitative and qualitative approaches. They offer examples of how gender equality is connected with different sexual and reproductive health issues, such as condom use, adolescent pregnancies, maternal-child health, and sexual health. MacPherson et al. chart the scenario by presenting a critical overview of gender equity and sexual and reproductive health in Eastern and Southern Africa. They conclude that sexual and reproductive health is central to gender equity in health in the region, and that interventions to improve it have to be enacted not only within the health system but also outside the system (10). In the field of maternal and child health, the paper by Mason et al. contributes not only to the visibilisation of the importance of maternal nutrition to improve a newborns’ health but also that of women's health (11). In the field of young people's sexual and reproductive health, Mehra et al. explore gender differences in the association between condom efficacy and condom use among Uganda university students. They show that women are at higher risk of inconsistent condom use, and relate these findings with gender–power relations, proposing that the feminisation of the HIV epidemic in this setting could be driven by gender inequalities (12). Christofides et al. present a longitudinal study with teenage girls in South Africa, exploring the relationship between gender inequality and gender-based violence and subsequent unplanned and unwanted pregnancies. They found that although some of the measures of gender inequality were not associated with unplanned and unwanted pregnancies, the role of gender power was evident in that teenage girls who experienced physical violence were more likely to have an unwanted pregnancy (13). Finally, in the field of sexual health, DeMeyer et al. offer evidence regarding the strong link between gender equality and sexual health. Their cross-sectional study with young people in Bolivia and Ecuador reveals that more egalitarian gender attitudes are related to higher current use of contraceptives within the couple, more positive experiences and ideas about sexual intercourse, and better communication about sexuality with the partner among sexually active and sexually non-active adolescents (14).

*Violence against women or intimate partner violence* (IPV) is addressed in three articles. Women's lived experiences of coping with domestic violence in rural Indonesia is described by Hayati et al. as an ‘elastic band strategy’, meaning a long-term process of moving between positions of opposing the violence and accepting it. The interviewed women faced lack of institutional support (15). Edin and Nilsson highlight the specific circumstances of living in violent relations and becoming pregnant. The study is based in Sweden and they conclude that Swedish health care institutions and maternal care need to become more aware of the way pregnant women exposed to IPV express their situation, which often is indirect and difficult to understand (16). In a cross-sectional survey, Burgos-Soto et al. investigated lifetime prevalence of physical and sexual violence among HIV-infected women in Togo compared to non-infected women. The prevalence was significantly higher among infected women (17).

Five articles deal with questions about *men*, *masculinities*, *and health*. They concern traditional masculinities of dominance and power as well as emerging, new forms of masculinities, of which the former are regarded to be detrimental to both men's and women's health. In the Nicaraguan context, Torres et al., have investigated young men's struggle for more gender-equitable masculinities and they conclude that the emerging forms of masculinities found within the study can help improve gender relations and that they might be labelled ‘health-promoting masculinities’ (3). From the Ecuadorian context, Goicolea et al. investigate how young men understand IPV. The main finding is that the young men take a stance in which they condemn violence whereas at the same time they do not really reject sexism (18). In a study from southern Spain, Marcos Marcos et al. provide insights into constructions of masculinities that are dependent on collective practices and performative acts which have a bearing on health behaviour and gender equality (2). In a study on black South African men's constructions of respect and masculine identities in regard to violence and HIV, Gibbs et al. suggest ways of working with men in order to reduce risky behaviours and prevent violence (1). In a study from Thailand on men's experiences of alcohol addiction and treatment, Hanpatchaiyakul et al. found three clusters of experiences as ways of describing the development of addiction. They emphasise the importance of addressing concepts of masculinity and hegemony in relation to treatment of alcohol addiction among Thai men (19).

Two papers focus on *epidemiological perspectives* on gender and health. Malmusi et al. use data from the population living conditions survey in Catalonia, Spain, to explore if unequal gender distribution of resources can explain women's poorer self-rated health across social classes. After adjustment for individual income, they found that the association between sex and self-rated health was eliminated, and especially so for the manual classes. Thus, individual income accounted for the observed health inequalities by gender and social class. Malmusi et al. stress the need for policies to close the gender pay gap and to facilitate women's labour participation in order to reduce gender inequalities in health (20). Bonita and Beaglehole discuss gender bias in the global discourse on health, which focuses on women's reproductive capacity and neglects the influence of non-communicable diseases (NCDs) on women's health. This neglect may result in women receiving fewer examinations and diagnosis tests, despite the fact that the absolute numbers of NCD deaths in women are similar to that of men. Bonita and Beaglehole propose that women and NCDs should be prioritised on the post-2015 sustainable human development agenda (21).

One article addresses issues of *access to health services* from a gender perspective. Otero-García et al. explore rural midwifes’ perceptions on immigrant women's access to sexual and reproductive health services. According to Otero-Garcia et al., midwifes relate underutilisation of such services by immigrant women to gender inequalities and access barriers (22).

*Health policy* is discussed in two of the papers. In their commentary, Himabindu et al. discuss how the worldwide attention to the ‘rape crisis’ in India generated widespread political support for strengthening legal responses towards violent crimes against women. Despite this, gender-based violence remains a vast problem in India, due to the deep-rooted patriarchy of Indian society making laws and regulations not enough as a solution. According to Himabindu et al., the portrayal of women in the Indian cinema plays a significant role in reconstructing prejudicial attitudes towards women. They call upon health workers and researchers to take the lead in shaping a social response towards gender violence by applying a gender lens to their work and striving for the empowerment of women (23). Gavriilidis et al. report from an evaluation of a gender equity integration development plan (GEIPD) in the city of Malmö, Sweden, aiming to increase gender equity in all aspects of city life. They applied a policy empowerment index to understand how policy planning can affect constituent empowerment. Gavriilidis et al. found that 50–90% of Malmö residents were concerned with gender inequality at home or at work, despite living in one of the most gender equal countries in the world. Their evaluation showed that the GEIPD has a strong potential to empower its constituency, with its strong emphasis on protection against gender discrimination in employment, education, and distribution of resources and agency (24).

To conclude, we argue that there are some important gaps that need to be addressed in future research dealing with gender and health. Work-related health is considered to become one of the leading causes of ill-health in the world (25). For instance, work is heavily gendered in a number of ways. There is substantial gender segregation and discrimination of women in terms of income, career opportunities, and access to leading positions. In unpaid work, women usually take a greater responsibility for reproductive work in child care, care for the elderly, and household duties. Women report more stress at work and work-related burnout is common. This calls for health researchers to address issues of work-related ill-health, including unpaid work, so that the total work load is scrutinised. Health problems related to climate change have up until now not focused much on gender, and the risk of overlooking gendered outcomes of global warming, car driving, transportation, and so on, is obvious here (26). We also welcome a development of postcolonial perspectives into gender and health research. Postcolonial theory has as yet mainly been developed in social science and cultural studies. The need for a theoretical integration of such perspectives is great, and would highlight inequalities, disparities, and tensions between the Global South and North in terms of public health policy and international declarations (27). Gendered effects of international migration as well as the vast demands on societies and individuals in terms of ageing populations around the world are other issues of importance for future global health research on gender and health.

*Ann Öhman* Division of Epidemiology and Global Health Department of Public Health and Clinical Medicine Umeå Centre for Global Health Research Umeå University, Umeå, Sweden Umeå Centre for Gender Studies Umeå University, Umeå, Sweden ann.ohman@ucgs.umu.se *Malin Eriksson* Division of Epidemiology and Global Health Department of Public Health and Clinical Medicine Umeå Centre for Global Health Research Umeå University, Umeå, Sweden *Isabel Goicolea* Division of Epidemiology and Global Health Department of Public Health and Clinical Medicine Umeå Centre for Global Health Research Umeå University, Umeå, Sweden
